# The p140Cap adaptor protein as a molecular hub to block cancer aggressiveness

**DOI:** 10.1007/s00018-020-03666-w

**Published:** 2020-10-20

**Authors:** Vincenzo Salemme, Costanza Angelini, Jennifer Chapelle, Giorgia Centonze, Dora Natalini, Alessandro Morellato, Daniela Taverna, Emilia Turco, Ugo Ala, Paola Defilippi

**Affiliations:** 1 Department of Molecular Biotechnology and Health Science, Università degli Studi di Torino, Via Nizza 52, 10126 Torino, Italy; 2 Department of Veterinary Sciences, Università degli Studi di Torino, Largo Paolo Braccini 2, 10095 Grugliasco, TO Italy

**Keywords:** *SRCIN1*, Metastasis, Chromosome 17q12, Gene amplification, miRNA, Interactome analysis

## Abstract

The p140Cap adaptor protein is a scaffold molecule encoded by the *SRCIN1* gene, which is physiologically expressed in several epithelial tissues and in the neurons. However, p140Cap is also strongly expressed in a significant subset of cancers including breast cancer and neuroblastoma. Notably, cancer patients with high p140Cap expression in their primary tumors have a lower probability of developing a distant event and *ERBB2*-positive breast cancer sufferers show better survival. In neuroblastoma patients, *SRCIN1* mRNA levels represent an independent risk factor, which is inversely correlated to disease aggressiveness. Consistent with clinical data, *SRCIN1* gain or loss of function mouse models demonstrated that p140Cap may affect tumor growth and metastasis formation by controlling the signaling pathways involved in tumorigenesis and metastatic features. This study reviews data showing the relevance of *SRCIN1*/p140Cap in cancer patients, the impact of *SRCIN1* status on p140Cap expression, the specific mechanisms through which p140Cap can limit cancer progression, the molecular functions regulated by p140Cap, along with the p140Cap interactome, to unveil its key role for patient stratification in clinics.

## Introduction

p140Cap, also known as p130Cas-associated protein or SNIP (SNAP-25 Interacting Protein), is a scaffold molecule involved in the formation of multi-protein complexes [1–4]. The p140Cap protein is encoded by the Src Kinase Signaling Inhibitor 1 gene (*SRCIN1*), located on chromosome 17q12 [4]. Structurally, p140Cap is a highly hydrophilic protein, which does contain neither a signal sequence nor a putative transmembrane domain [1]. Its amino acid sequence includes a tyrosine-rich domain, a putative actin-binding site, two proline-rich domains containing multiple PPXY and PXXP involved in protein–protein interactions with the SH3 domains, a coil-coiled region and two C-terminal highly charged regions [1, 3]. Furthermore, p140Cap includes several serine, threonine and tyrosine residues that upon phosphorylation could serve as binding sites for proteins involved in the signaling network [5]. In the human, p140Cap shares up to 60% homology and 40% identity (when aligned with BLASTp) only with the Sickle-Tail (SKT) protein, the latter encoded by the human KIAA1217 gene [6, 7]. The p140Cap protein is physiologically highly expressed in the cerebral cortex and in the cerebellum, and poorly expressed in several epithelial tissues. Indeed, p140Cap was first discovered as an interactor of SNAP-25 in the rat brain [1]. Its role in neurons was further evidenced by several groups, on account of the use of the p140Cap knock-out mouse model [8–10]. Subsequently, the human orthologue of rat SNIP proteins was identified by mass spectrometry in epithelial cells as the 140 kDa KIAA1684 protein indirectly associated with p130Cas through the last 217 amino acids of p140Cap and the amino acids 544–678 of p130Cas [3]. Indeed, the p140Cap C-terminal region (amino acids 1000–1217) directly binds to the SH3 domain of Src kinase, which in turn directly binds to p130Cas [11]. Despite these data, p140Cap involvement in tissues other than the brain has not been deeply investigated to date. This study reviews the expanding amount of data on the p140Cap role in human cancer published following our previous review [2]. Overall, these data underline the fact that a positive p140Cap status correlates with increased cancer patient survival. Therefore, the genomic status of *SRCIN1* gene and the role of its post-transcriptional regulation in cancer are illustrated. Moreover, both the molecular mechanisms controlled by p140Cap and the functional features of p140Cap tumor-expressing cells are underlined as well as its scaffold role based on the analysis of specific binding proteins and of the interactome in cancer cells.

### Genomic organization of the *SRCIN1* gene

The human *SRCIN1* gene which consists of 27 exons is located on chromosome 17q12, and is highly conserved in mammals, especially in primates but also in mice, rats, dogs, and cows [4]. At least six different coding transcripts are reported as deriving from the *SRCIN1* gene in the Ensemble database. Furthermore, human *SRCIN1*-flanking regions contain several genes involved in tumor initiation and progression, such as *ERBB2* (17q12), *BRCA1* (17q21), retinoic acid receptor-α (*RARA*; 17q21) and signal transducer and activator of transcription 3 (*STAT3*; 17q21). These genes are often amplified or undergo a gain of function role in human tumors, suggesting that *SRCIN1* might also be subjected to such chromosome rearrangements [12]. Therefore, the *SRCIN1* gene-containing region is a typical example of a synteny block that shares a common order of homologous genes between human and mouse genomes. Moreover, the structure of human and mouse *SRCIN1* genes are highly comparable [3]. Data on p140Cap gene expression regulation are currently limited, in that, amplification or rearrangements can occur in this region but additional epigenetic mechanisms can also account for altered p140Cap protein expression in cancer (see below).

### *SRCIN1*/p140Cap physiological expression

mRNA expression data for 37 different normal tissues obtained from RNA deep sequencing, as provided by the Human Protein Atlas (HPA) database, https://www.proteinatlas.org/ENSG00000277363-SRCIN1/tissue, show high expression in the brain, the salivary glands and the skin. Protein expression data for 44 normal human tissues, based on immunohistochemistry (IHC) profiling, showed that p140Cap is highly expressed in the brain and in the testes, while it is barely expressed in the thyroid and the parathyroid glands, the salivary glands, the pancreas, the nasopharynx, the bronchi and the lungs, the kidneys, the fallopian tubes, the breast, the heart muscle and smooth muscle. p140Cap expression in different healthy tissues versus tumor counterparts have also been analyzed in the studies which report its post-transcriptional regulation by miRNAs (see below).

Furthermore, in the pancreas of Wistar rats, p140Cap has been found enriched in beta cells by IHC, revealing a cytoplasmic granular pattern [13]. Instead, in normal human breast tissues deriving from reduction mammoplasty by IHC, a selective expression of p140Cap has been detected in the alveolar luminal cells, whereas no staining was visible in ductal epithelial or myoepithelial cells [14]. Interestingly, the characterization of the MMTV-p140Cap transgenic mouse model, overexpressing the p140Cap cDNA under the MMTV promoter in the mammary gland, showed that p140Cap overexpression does not affect mammary gland development and differentiation but only induces a slight delay in post weaning lobular involution [15]. Further investigations are of course necessary to better understand the role of p140Cap in mammary gland physiology. A more detailed analysis of p140Cap expression and function in physiological conditions is available for the brain. p140Cap is abundantly expressed in the cerebellum and the telencephalon, including the hippocampus, neocortex, entorhinal cortex, visual cortex [16] and in the nucleus accumbens [17, 18]. p140Cap subcellular localization in neurons has been detected at the synaptic level, both in pre- and postsynaptic compartments [16, 19]. In the post-synapse, p140Cap plays a key role in actin remodeling and in the regulation of dendritic spine morphology besides acting as a hub for the formation of postsynaptic complexes following the interaction with various proteins, underlying a potential role in these compartments [8, 9, 20, 21].

### p140Cap and its binding partners

#### p140Cap protein structure and modifications

**Fig. 1:**
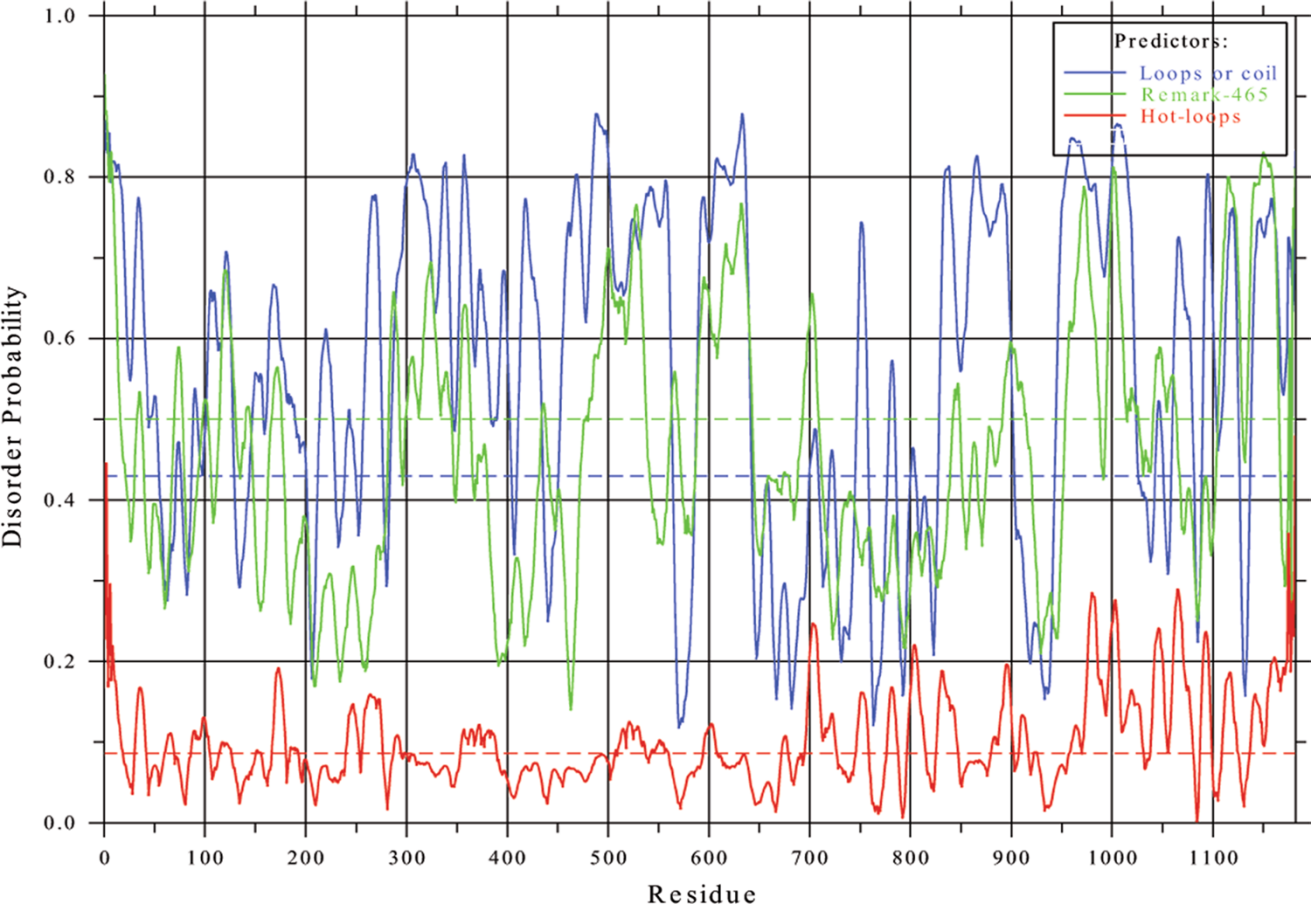
p140Cap and intrinsic disorder. Disorder probability of p140Cap human amino acid sequence (sequence ID: NP_079524.2), with DisEMBL computational tool. Figure downloaded from: DisEMBL https://dis.embl.de/, sequence ID: NP_079524, Intrinsic Protein Disorder Prediction 1.52). Predictions are shown according to each of the three different criteria to define the disorder level of protein residues, namely: loops or coils (blue line): loop assignment can be used as a necessary but not sufficient requirement for a disorder; -hot loops (red lines): constitute a subset of loops having a high degree of mobility as determined by C-alpha temperature (B-) factors. It follows that highly dynamic loops should be considered a protein disorder; missing coordinates in X-ray structure as defined by REMARK465 entries in PDB (green lines). Non-assigned electron densities in X-ray 3D structures most often reflect intrinsic disorder, and can be used in disorder predictions. For more detail, see [24]. The predicted probabilities are shown as curves along the sequences; scores should be compared to the corresponding random expectation value (dotted lines)

p140Cap shares some features with the “Intrinsic Disorder Proteins” (IDPs) containing Intrinsically Disordered Regions (IDRs), which are amino acid sequences which cannot fold spontaneously into stable, well-defined globular three-dimensional structures and often remain disordered and fluctuate rapidly from coils to collapsed globules [22, 23], as evidenced by the Phyre2 database (Protein Homology/analogY Recognition Engine V 2.0), which revealed 28% alpha helices, 2% beta strands and 75–77% disordered sequences. Consistently, similar features were confirmed downloading data from the Intrinsic Protein Disorder Prediction web server [24] DisEMBL https://dis.embl.de/, sequence ID: NP_079524.2). Figure 1 confirms the presence of many IDRs, especially in the C-terminal region, and in the region between amino acids 300 and 600, according to three different criteria (loops/coils, hot loops and missing coordinates) used as prediction parameters. Scaffold proteins selectively bring together specific proteins within signaling pathways to facilitate and promote interactions between them [25]. In this context, mainly disordered regions of p140Cap could allow interactions with alternative binding partners to promote specific interactions among signaling proteins.

**Fig. 2:**
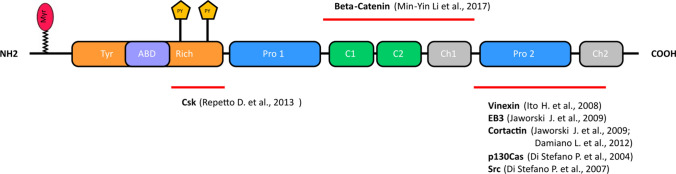
p140Cap structure and main interactors. p140Cap consists of an N-terminal tyrosine-rich region (Tyr-rich), an actin-binding domain (ABD), a proline rich domain (Pro1), a coil-coiled region (C1-C2), two domains rich in charged amino acids (CH1, CH2) and a C-terminal proline-rich domain (Pro2). Lipidation (Myristoylation, Myr) and functional tyrosine phosphorylation (PY) EPLYA and EGLYA are show. The binding regions of Csk, β-Catenin, Vinexin, EB3, Cortactin, p130Cas and Src are shown by the red lines

The in vivo phosphorylation of p140Cap was assessed by phosphorylation-directed multistage tandem mass spectrometry (pdMS^3^), in human breast cancer cells [5]. The analysis revealed the presence of three peptides containing phosphorylated serine residues (namely RGpSDELTVPR, RFpSNVGLVHTSER, and TEKPSKpSPPPPPPR). Among them, serine S45 and S987 are conserved in human and mouse p140Cap sequences and have been previously identified in murine phosphoproteomic screening investigations (https://www.uniprot.org/uniprot/Q9QWI6), suggesting the relevance of these phosphorylation sites across species, even if their functional role has not yet been unraveled [5]. pdMS^3^ analysis of p140Cap also revealed the presence of tyrosine phosphorylated residues, consistent with previous data showing that p140Cap is phosphorylated on tyrosine residues upon integrin-mediated adhesion or EGF receptor activation in epithelial cells [3]. In particular, one tyrosine residue in the sequence 392-GEGLpYADPYGLLHEGR-407 (briefly called EGL**Y**A) has also been found phosphorylated in the murine brain by a large-scale identification assay for tyrosine phosphorylation sites [26]. Noteworthy is the fact that p140Cap also harbors another sequence, similar to EGL**Y**A, which is EPL**Y**A (Y264). Both “EPLYA” and “EGLYA” tyrosine residue-containing sequences are conserved in humans, mice and rats (https://www.phosphosite.org) and have been identified as the main sites for tyrosine phosphorylation [5]. In addition, p140Cap ability to act as a negative regulator of cell migration and proliferation mainly resides in these phosphorylated tyrosine residues [27]. Mechanistically, these two sequences were shown to be the major substrates for the Abelson (Abl) kinase, which controls actin remodeling, cell motility and adhesion and cytoskeleton dynamics [28, 29] in HEK-293 cells [5]. It is worth highlighting that both EPLYA and EGLYA sequences are analogous to the EPIYA motif, previously described in the bacterial CagA protein involved in Helicobacter Pylori pathogenesis [30], where the phosphorylated CagA EPIYA sequence can associate with Csk SH2 domain, resulting in Csk membrane recruitment with subsequent inhibition of Src Family Kinases (SFK) [31, 32]. The EPIYA motif has similar functions in the mammalian Pragmin/SgK223 protein [32, 33]. Consistently with these data, the tyrosine included in both EGLYA and EPLYA sequences in p140Cap plays a crucial role in p140Cap interaction with the Csk kinase [5] (see Fig. 2). In this scenario, p140Cap takes part in a macromolecular complex activating Csk consequently leading to inhibition of Src and downstream signaling as well as cell motility and invasion [11]. Thus, expression of proteins such as p140Cap or Pragmin, which contain EPIYA-like motifs, could interfere with Csk activation [34] and/or localization [33], finely tuning SFK activity inside the cells.

#### p140Cap-interacting proteins

In addition to Src and Csk, p140Cap also binds to Beta-catenin through the 351–1051 amino-acid portion [10]. The terminal proline-rich domain of p140Cap associates with Vinexin [16], and Cortactin, a Src kinase substrate and an F-actin-binding protein [8, 35]. A short 92 amino acid C-terminal region (aa 1124–1216) also directly interacts with EB3, a member of the microtubule plus-end tracking protein EB family in the brain [8]. p140Cap has also been found in interactome studies performed both in mouse and human samples (BioGRID https://thebiogrid.org/207768/ and https://thebiogrid.org/123275/ respectively). p140Cap was found specifically present in interactome of PPP1R9B (protein phosphatase 1 regulatory subunit 9B) [24], SNCA (synuclein, alpha) [36], YWHAE (tyrosine 3-monooxygenase/tryptophan 5-monooxygenase activation protein) [37], LNX1 (ligand of numb-protein X1) [38] and AGAP2 (ArfGAP with GTPase domain) [39], from mouse samples. p140Cap was also identified in the interactome of NUFIP1 (nuclear fragile X mental retardation protein-interacting protein) [40, 41], BCAS4 (breast carcinoma amplified sequence 4) [41], ESR2 (estrogen receptor 2) [42], from human samples.

### *SRCIN1*/p140Cap expression in human tumors correlates with good prognosis

**Fig. 3:**
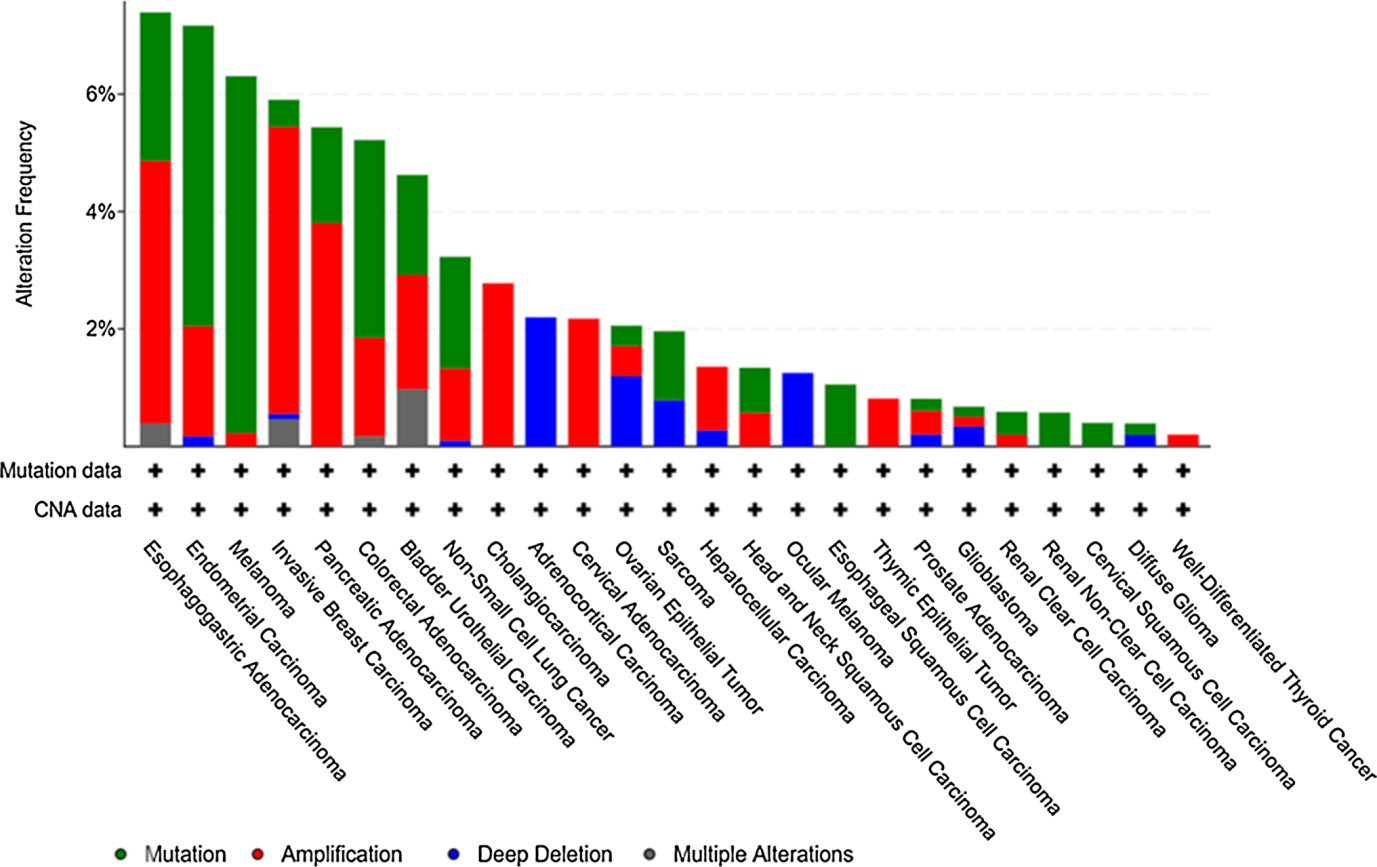
Alteration frequencies of *SRCIN1* gene in cancer. Frequencies of SRCIN1 gene alterations in various types of cancer included in the TCGA PanCancer Atlas Studies. Mutations, deep deletions, amplifications and multiple alterations are shown in different colors. Figure download from: cBioPortal for Cancer Genomics (https://www.cbioportal.org/)

The *SRCIN1* gene status in cancer has been downloaded as shown in Fig. 3, from the *cBioPortal* for Cancer Genomics, https://www.cbioportal.org, which provides visualization, analysis and download of large-scale cancer genomics datasets, including the TCGA repository. Figure 3 groups the tumors in which *SRCIN1* has been found as amplified (red), mutated (green) or deleted (blue), indicating that the *SRCIN1* gene can undergo several alterations in many cancer cohorts, including breast cancer.

**Fig. 4:**
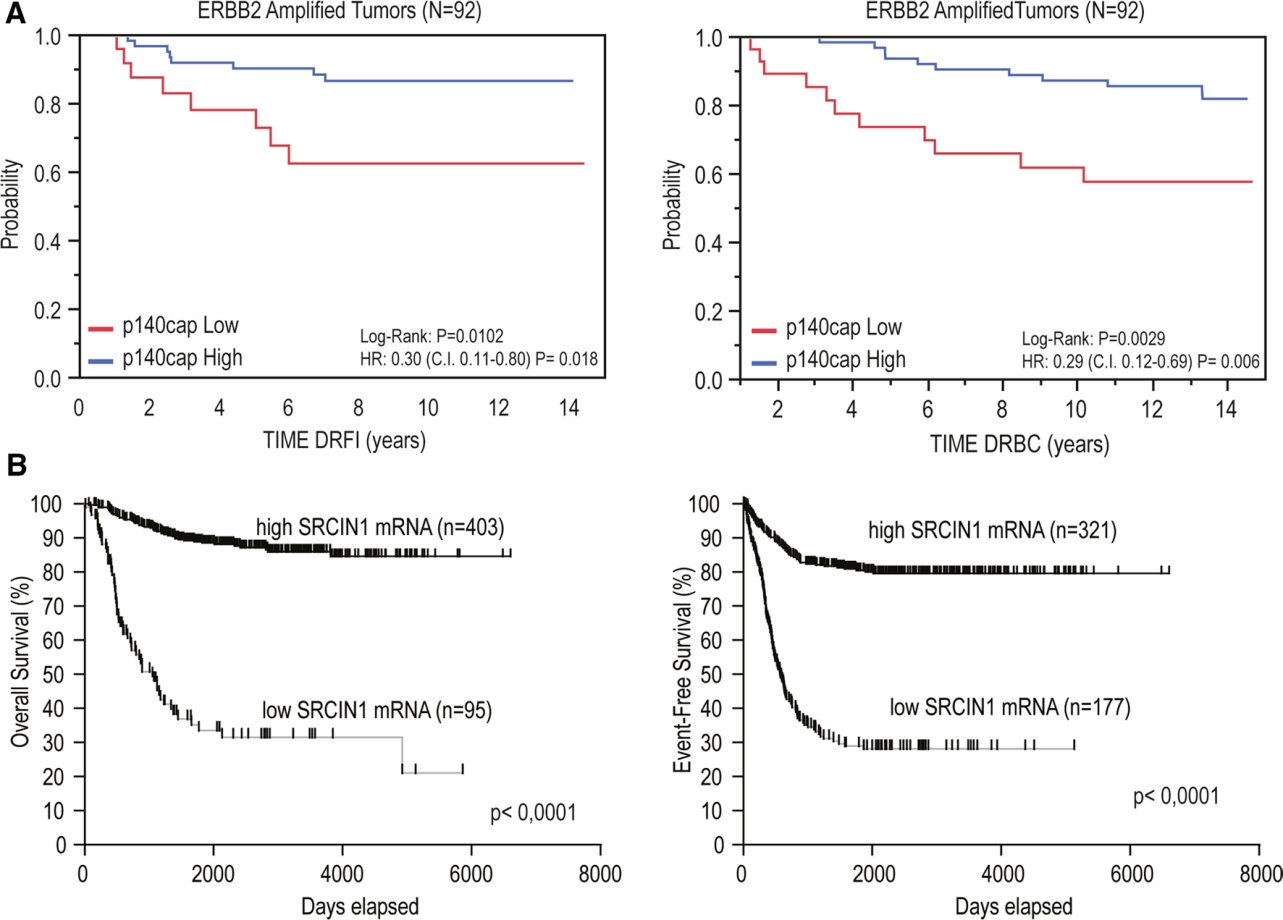
p140Cap expression in cancer patients correlates with good prognosis. **a** Kaplan–Meier curves for DRFI (left panel), and DRBC (right panel) stratified by p140Cap IHC expression in a cohort of 92 ERBB2-positive patients [15]. **b** Kaplan–Meier curves for overall (left panel) and event-free (right panel) survival stratified by SRCIN1 expression in a cohort of 498 NB patients [47]

*(A) Breast Cancer*. The likelihood that expression and genomic profiling of *SRCIN1*/p140Cap might impact on the disease was first evaluated in human breast cancer (BC), one of the most common cancers with more than 2 million new cases in 2018 and 450,000 deaths each year worldwide [43, 44]. Clinically, BC is classified into three basic therapeutic groups as follows: estrogen-receptor (ER) positive, ERBB2 (also called HER2)-positive, and triple-negative, lacking expression of ER, progesterone-receptor (PgR) and ERBB2 [43]. In BC, p140Cap expression was linked to a less aggressive disease [14]. Interestingly, 94.8% aggressive G3 tumors, 87% node positive, 86.5% tumors with a high mitotic count, and 76% highly proliferative tumors (revealed by the Ki67 staining marker) lose p140Cap expression, indicating that the most aggressive BCs do not express p140Cap, showing an inverse correlation with malignancy. Moreover, expression of p140Cap on a consecutive cohort of 622 invasive BCs showed that positive p140Cap status (with an IHC score ≥ 1) is associated with good prognosis markers, such as negative lymph node status, ER and PgR-positive status, small tumor size, low grade, low proliferative status, and ERBB2-positive status. Positive p140Cap status is also associated with BC molecular subtypes, being expressed in more than 85% Luminal A, 77% Luminal B and only 56% Triple-Negative tumors [15]. In the subgroup of *ERBB2*-amplified BC, a high p140Cap status predicts a significantly lower probability of developing a distant event and of death from BC (Fig. 4, panel a). By contrast, no significant difference is observed in patients who did not harbor *ERBB2* amplification. In conclusion, p140Cap expression is associated with reduced risk of metastasis (and death from cancer), in the *ERBB2*-amplified subgroup of BCs, suggesting a possible role of p140Cap in counteracting the migratory and/or metastatic ability of *ERBB2*-amplified cancer cells.

*(B) Neuroblastoma*. In addition to BC, the relevance of p140Cap has been deeply investigated in Neuroblastoma (NB), a complex disease with different outcomes and responsible for a large proportion of deaths due to cancer in childhood [45, 46]. Primary NBs originate from genetic abnormalities in the neural crest-derived sympathoadrenal cells. Bearing in mind the functional role of p140Cap in differentiated neural cells [8, 9], p140Cap was detected by IHC in the medulla of normal human neonatal adrenal glands [47], indicating that p140Cap is expressed in the main site of origin of NBs. The Kaplan–Meier analysis of *SRCIN1* gene expression on the R2 platform, containing gene expression profiles and clinical information of 498 NB cases compared to overall survival (OS) and event-free survival (EFS), showed that high *SRCIN1* expression was significantly associated with good prognosis (403 patients) whereas low expression was observed in 95 poor prognosis patients (Fig. 4, panel b). Moreover, high *SRCIN1* mRNA expression was found in secondary event-free survival (321 patients) while a low expression was significantly associated with reduced secondary event-free survival (177 patients) leading to the conclusion that *SRCIN1* is a prognostic risk factor for NB. The International Neuroblastoma Staging System (INSS) defines 5 stages of NB progression [48], from Stages 1 and 2 (localized tumor, without lymph node involvement) to Stage 3 and Stage 4 (metastatic disease, with dissemination in distant organs). Stage 4S specifies that in children 1-year old or younger, a metastatic disease which may undergo spontaneous regression is usually associated with 90% survival rate at 5 years. Stage (st) 4 tumors have a significantly lower expression of *SRCIN1* mRNA relative to st1, st2, st3, st4s groups, supporting the conclusion that *SRCIN1* mRNA expression is higher in patients with low metastatic spread. Moreover, *SRCIN1* mRNA expression is a favorable prognostic factor, both in terms of OS and EFS, regardless of the other known risk factors, including *MYCN* amplification, INSS stage, and age at diagnosis. Overall, to date, p140Cap expression by IHC on NB samples has not been studied owing to lack of available cancer tissues, but *SRCIN1* mRNA expression correlates with a good outcome and is an independent prognostic marker for NB [47].

### Gene status and regulation of expression of *SRCIN1* in cancer

*(A) The SRCIN1 and ERBB2 genes are co-amplified in a subset of human breast tumors*. As shown above, the *SRCIN1* gene, located on Chromosome 17q12, is 1 million base pair centromeric to the ERBB2 gene. BAC array Comparative Genomic Hybridization (aCGH) on 200 *ERBB2-*amplified tumors revealed that *SRCIN1* gene is altered in 70% of cases, with a copy number (CN) gain for *SRCIN1* in 61.5% of the cases [15]. Relevantly, a Kaplan–Meier analysis showed that *SRCIN1* amplification correlates with improved survival. Moreover, mRNA expression and *SRCIN1* gene copy number were significantly correlated in 50 out of 200 *ERBB2*-amplified tumors. Further, FISH analysis on an independent patient cohort showed that 56% of the *ERBB2*-amplified tumors specimens were also amplified for *SRCIN1*, while in ERBB2-negative BCs, *SRCIN1* CN was never altered [15]. Overall, the *SRCIN1* gene is frequently, but not necessarily, co-amplified with *ERBB2* in BCs, following chromosomal rearrangements which result in *ERBB2* amplification, thus contributing to the biological heterogeneity of this BC subgroup [49]. Moreover, for the first time, the correlation with the positive patient outcome outlines that a gene in the *ERBB2* amplicon may counteract ERBB2 oncogenic properties in BCs.

*(B) SRCIN1 gene status in Neuroblastoma*. The most frequent chromosome imbalance in all high stage NBs is the 17q gain, which occurs in 50–70% of cases [50, 51] and is associated with poor prognosis as an independent indicator of adverse outcome [52]. In 17 out of 225 NB patients with the 17q gain, the *SRCIN1* gene status is altered, suggesting putative modifications of *SRCIN1* gene expression in NB patients. However, the differences in OS between patients harboring these alterations does not reach statistical significance (*P* = 0.5), indicating that the size of the cohort should be increased. However, the presence of translocation breakpoints involving the 17q portion and interrupting the *SRCIN1* gene (heterozygous deletion or copy neutral-LOH) have been detected in primary tumors of all stages with 17q gain, which associates a poor prognosis with NB [47]. *SRCIN1* gene status is also affected in a panel of human NB cell lines. Indeed, *SRCIN1* is lost in ACN, a neuroblast-like cell line derived from bone marrow metastasis [53], and a single copy is found in SH-SY-5Y, IMR-32, and HTLA-230 cells, while function gain can be seen in LAN-1 cells, and subjected to copy neutral-LOH in SK-N-SH cells [47].

*(C) SRCIN1 post-transcriptional regulation by miRNAs*. MicroRNAs (miRNAs) are small non-coding RNAs which negatively regulate gene targets by affecting both mRNA stability or translation. Dysfunction of miRNAs is implicated in various human cancers and depending on their targeted genes, they can act as either oncogenes or tumor suppressor players [54, 55]. Recently, several papers have shown that p140Cap can be targeted by a variety of miRNAs in different types of tumors such as lung, pancreatic, gastric, breast, ovarian and colorectal cancer as well hepatocellular and cervical carcinomas (Table 1). In almost all the cases, the direct targeting of p140Cap has been validated in cancer cell lines and the resulting downregulation of p140Cap expression promotes tumor features such as proliferation [56–68], migration [56, 58, 60, 61, 63, 67, 68], invasion [60, 64] and angiogenesis [69]. Among the reported miRNAs, miR-150 has been described as a negative regulator of p140Cap in multiple malignancies such as BC, where it promotes migration, invasion and expression of EMT markers in cancer cells [70]. p140Cap protein levels are downregulated in cancer compared to adjacent non-cancer tissues and an opposite expression pattern has been observed for related miRNAs. Of note, high miR-371a expression is correlated with poor clinical pathological features of hepatocellular carcinoma, whereas decreased expression of p140Cap associates with adjacent organ invasion, microscopic vascular invasion, and advanced tumor stage [71]. Unlike p140Cap protein levels, mRNA abundance varies randomly in human lung cancer tissue samples, suggesting that miR-150 may regulate p140Cap by translational repression rather than by RNA degradation [56]. The increasing amount of data describing the inhibition of p140Cap expression by oncogenic miRNAs may lead to new therapeutic strategies that aim at restoring p140Cap levels and its tumor suppressor functions. However, additional efforts need to be made to better understand the relevance of p140Cap downregulation by miRNAs in vivo in preclinical studies.

**Table 1:** miRNAs targeting p140Cap

microRNAs	Type of cancer	References
miR-150	Lung cancer	[56] Cao et al. 2014, [66] Zhang et al. 2018
	Cervical carcinoma	[68] Zhu et al. 2019
	Breast cancer	[70] Lu et al. 2019
	Gastric cancer	[62] Quan et al. 2019
miR-374a	Gastric cancer	[64] Xu et al. 2015
	Pancreatic cancer	[61] Ma et al. 2019
miR-211	Lung cancer	[65] Ye et al. 2016
miR-873	Lung cancer	[58] Gao et al. 2015
miR-32	Hepatocellular carcinoma	[57] Chen et al. 2018
miR-181a	Colorectal cancer	[69] Sun et al. 2018
miR-371a	Hepatocellular carcinoma	[71] Bai et al. 2018
miR-510	Lung cancer	[63] Wu et al. 2019
miR-208a	Lung cancer	[60] Liu et al. 2019
miR-665	Ovarian cancer	[59] Li et al. 2019

### p140Cap functional role in tumors

The correlation data described above for BC and NB patients lead to the hypothesis that p140Cap may attenuate the intrinsic biological aggressiveness of these tumors. Overall, p140Cap is able to limit the in vitro migration and invasion, as well as the in vivo tumor growth and metastasis formation of BC and NB models.

*(A) Breast Cancer*. p140Cap can regulate actin cytoskeleton and can interfere with cell spreading on the extracellular matrix, during the early phases of cell adhesion of NIH3T3, ECV304 cells [3], and of MCF7 BC cells, mainly through its carboxy-terminal proline-rich region, involved in Src binding [11]. This defective cell adhesion may negatively impact on migration ad invasion, as reported in triple negative MDA-MB-231 BC cells [11], and in ERBB2-positive SKBR3 and MDA-MB-453 cell lines [15]. However, p140Cap can also affect in vivo tumor growth. Indeed, the double transgenic mice NeuT-p140Cap, obtained by crossing the MMTV-p140Cap transgenic mice with the MMTV-NeuT mice, show a significant delay in the appearance of the first tumor, associated with a significant decrease in the total tumor burden, compared to NeuT mice [15]. Local apoptotic events within tumor development may also account for the difference in total tumor burden. Indeed, upon apoptotic stimuli and in 3D Matrigel-Collagen cultures, p140Cap cells showed increased sensitivity to apoptosis. Moreover, the ability of p140Cap-expressing tumor cells to activate an apoptotic program allows the cells to partially revert to a normal mammary tissue morphogenesis with the formation of an internal lumen, typical of normal mammary epithelial cells [15, 72]. The ability of p140Cap in counteracting cancer cell invasion of secondary sites can be accounted for owing to the combination of impairment of cell adhesion, migration and proliferation which is confirmed in both experimental and spontaneous metastasis assays [15, 35].

*(B) Neuroblastoma*. In NB cell lines, p140Cap affects in vitro migration and soft-agar growth, while, it correlates with a significant reduction in growth in in vivo xenografts, which is also confirmed by decreased IHC staining of the Ki67 proliferation marker, and a reduced number of spontaneous lung metastases [47]. It is to be pointed out that the quantification of several vascular markers highlighted a higher pericyte coverage, suggesting that p140Cap can impact on in vitro NB tumorigenic traits also by increasing tumor vessel stabilization.

## p140Cap-dependent signaling in tumors

*(A) Breast cancer.* p140Cap can control several signaling pathways in BC cells. The first to be identified was the down-regulation of the Src kinase activity, through the binding of the Csk protein, following cell matrix-adhesion or EGF stimulation [11, 19]. Upon association with p140Cap, Csk becomes activated and phosphorylates the Src Carboxy Terminal inhibitory tyrosine residue 527, which is enabled to associate the Src Amino Terminal SH2 domain, closing the active conformation of the molecule to an inactive state. As a consequence, p140Cap inhibits the downstream Src-dependent phosphorylation of the Focal Adhesion Kinase (Fak), tyrosine 925, impairing the stability of Fak and Src association, and of the p130Cas adaptor protein [11].

In addition, p140Cap affects Rac1 GTPase activity [11]. Rac-specific GEFs, such as Dock, Tiam1 and PRex1, have also been shown to play a relevant role in BC [73, 74]. ERBB2-positive p140Cap tumor cells show a significant decrease in the activation of Tiam1 and of Rac [15]. The amino terminal region of p140Cap (1–287 amino acids) is sufficient for the association of full-length Tiam1, and the truncated catalytic domain of Tiam1 with a concomitant decrease in the Tiam1 activity. Moreover, in a large cohort of Her2-positive breast cancer patients, high levels of *SRCIN1* expression positively correlates with increased survival in patients with high *TIAM1* expression [75]. These data also provide evidence of a new molecular complex made up of p140Cap, Tiam1 and E-cadherin at the cell membrane of ERBB2-positive tumor cells [75]. E-cadherin levels are highly down-regulated during the EMT (epithelial to mesenchymal transition) which controls the metastatic process [76, 77]. p140 tumor cells display up-regulation of both E-cadherin mRNA and cell surface protein levels [15]. The increased amount of immobilized E-cadherin at the cell surface relies on the impact of p140Cap on Csk/Src kinase mutual regulation [14]. Furthermore, p140Cap exerts an overall inhibitory effect on counteracting the EMT invasive program of ERBB2 tumors, as shown by a marked down-regulation of the EMT transcription factors Snail, Slug and Zeb1 of an EMT transcription program, and of the mesenchymal cell–cell adhesion protein N-cadherin [15]. Therefore, in addition to limiting Tiam1 activity, p140Cap could indeed contribute to strengthening the adherence junction stability through the stabilization of E-cadherin expression. Future studies will determine the way in which p140Cap participates in these molecular interactions in a spatial and temporal manner and affects E-cadherin junction stability, which is a key step in regulating tumor progression.

*(B) Neuroblastoma.* In ACN cells, p140Cap negatively regulates Src kinase activation as well as tyrosine phosphorylation of p130Cas, along with decreased phosphorylation of both STAT3 Tyr 705 (pSTAT3) and its upstream tyrosine kinase JAK2 [47]. Consistently, p140Cap silencing results in increased phosphorylation of Src and STAT3 in SH-SY-5Y, another well-characterized NB cell model. Interestingly, the overexpression of Src kinase has been associated with poor outcomes in NB patients [78], while its inhibition results in decreased proliferation and enhanced apoptosis in NB cells [79, 80]. Moreover, STAT3 can exert a pro-survival role in NB [81–83]. In line with this, p140Cap cells has shown decreased levels of the survival marker Bcl2 as well as increased Annexin V staining, after anoikis [47].

The increased survival of p140Cap-expressing NB patients could be explained as there being increased cell sensitivity to chemotherapy. Interestingly, p140 NB cells has shown significantly increased sensitivity to low doses (10, 100 nM) of doxorubicin and etoposide, two drugs used in NB first-line treatments. Consistently, in SH-SY-5Y cells, p140Cap silencing results in increased viability to both drugs. Both etoposide and doxorubicin prevent repair of the DNA strands, stopping the process of replication. In line with this, p140Cap NB cells harbor a significant increase foci/cell number in phosphorylated histone H2AX gamma, an established marker of DNA damage [84]. Overall, p140Cap NB cells display a significant decrease in cell viability to chemotherapy drugs, with an increased sensitivity to drug-dependent DNA damage. Furthermore, sensitivity of NB cells to doxorubicin and etoposide is additionally enhanced when used in combination with Saracatinib and Sugen, two well-known Src inhibitors. Combined regimens acted synergistically both in control and p140Cap-expressing NB cells; however, p140Cap cells has shown further increased sensitivity to the Src inhibitors in combined treatments, suggesting that chemotherapy and Src inhibitor combination synergistically decreases NB cell viability and this effect can be further increased by p140Cap expression. In addition to Src activity downregulation, p140Cap ability to associate with proteins belonging to multiple pathways can contribute to the increased sensitivity of p140Cap NB cells to combined treatments.

### p140Cap interactome in breast cancer and related signaling pathways

The assembly of multi-protein complexes (interactomes) is essential for carrying out basic biological functions, such as cell migration and proliferation, in which protein–protein interactions are built around adaptor proteins, at the plasma membrane level, in the cytoplasm, or in specific organelles. In healthy neuronal synapses, the molecular complexes and pathways underlying p140Cap function in pathological and physiological conditions have been interrogated using mass spectrometry (MS) combined with bioinformatics data and analyses. Interestingly, the p140Cap interactome in crude synaptosome revealed 351 p140Cap interactors which cluster to sub complexes mostly located in the postsynaptic density (PSD) [19]. The p140Cap interactome was also recently generated in ERBB2-positive BC cells [15, 85], leading to the identification of 373-interacting proteins. Consistent with the previously described role for p140Cap in cell–matrix and cell–cell adhesion, and with the already known interactors, the GO Ontology has shown significant enrichment for “Cell- substrate junction” and “Focal adhesion” terms [11, 14]. However, a previously unknown role for p140Cap complexes in protein homeostasis in BC emerges from the terms “Proteasome complex”, “Endopeptidase complex” and “Extrinsic component of plasma membrane”. In the enrichment analysis of GO Biological Process (BP) terms and in the Reactome pathway database, “Regulation of mRNA stability”, “Response to tumor necrosis factor” and other terms related to regulation of protein translation, DNA and RNA damage response, apoptosis and cell cycle were highly significant. It is worth noting that p140Cap interactome takes part in the “Wnt signaling pathway, planar cell polarity pathway”, a fundamental regulator of cell proliferation in cancer cells [86].

The Protein–Protein Interaction (PPI) Networks can be challenged by clustering algorithms and parameters to identify heterogeneous communities within the network, which often form ‘modules’ of proteins that functionally co-operate in specific pathways. Gene-disease and gene-functional annotation data can then be marked on those clusters to test functional/disease enrichment of the clusters. Within the p140Cap interactome, 15 communities were present, with a topology-functional relationship which allowed the identification of subsets of proteins which preferentially contribute to specific functions. For example, the Cluster C2 contains p140Cap and the tyrosine kinases Src and ERBB2, reinforcing the concept that p140Cap can associate and regulate tyrosine kinases [11, 87], which play key roles in BC transformation and progression.

## Conclusions and future perspectives

**Fig. 5:**
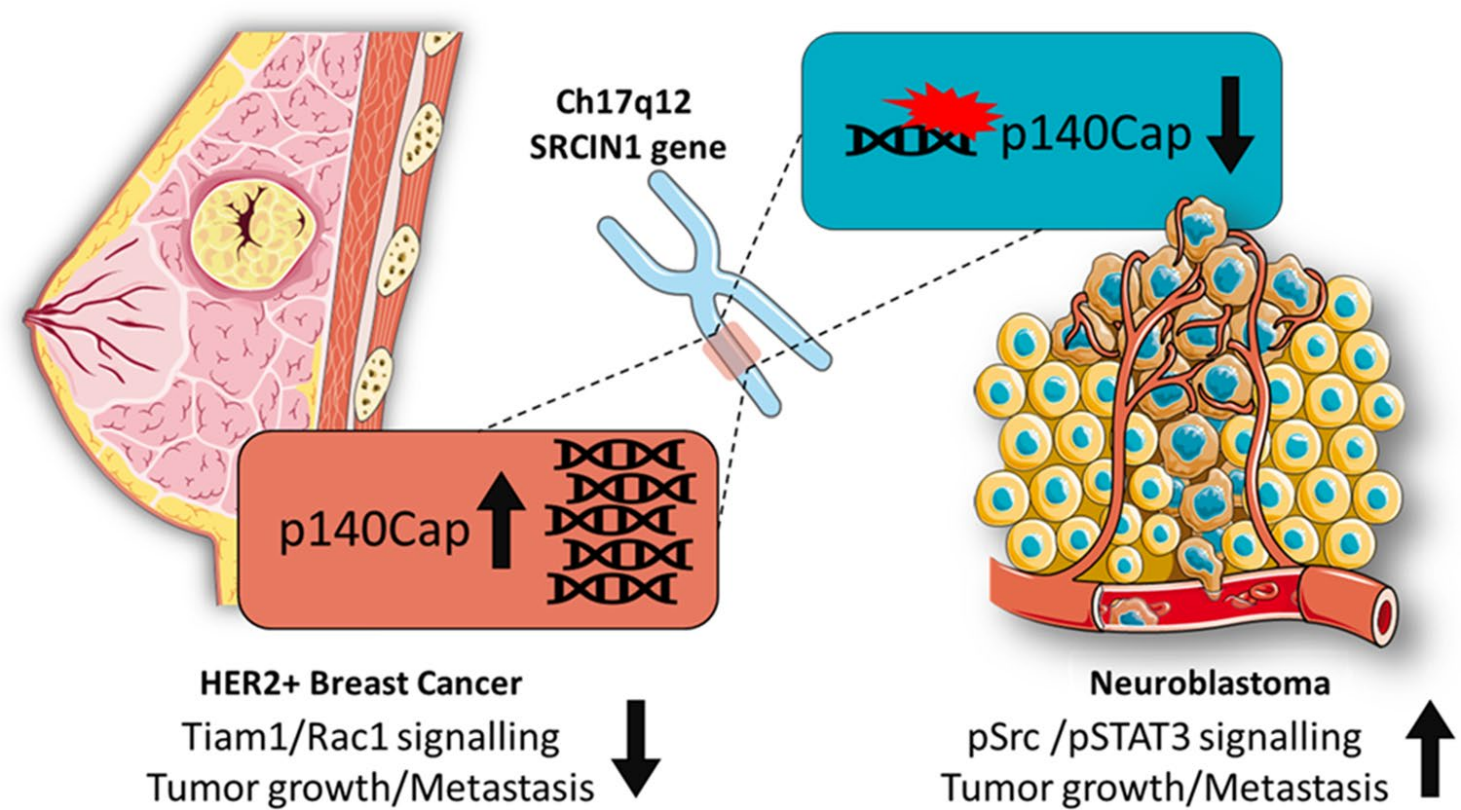
Impact of *SRCIN1*/p140Cap gene alteration in ERBB2/HER2-amplified breast cancer and neuroblastoma. Amplification of the *SRCIN1* gene in ERBB2/HER-2-positive breast cancer patients impairs tumor growth and metastasis mainly through the down-regulation of Tiam1/Rac1 axis. *SRCIN1* aberrant alterations such as translocations, deletions or loss of heterozygosity promote tumor growth, metastasis and drug resistance in NB patients, consistent with the loss of p140Cap expression, and inhibition of both Src and STAT3/Jak2 pathways

Based on the collected data, p140Cap shows tumor-suppressing properties, which oppose and interfere with cancer features in human malignancies. p140Cap expression correlates with a good prognosis and a delay in tumor progression in BC and NB patients (Fig. 5). However, further analysis is needed of *SRCIN1* gene status and p140Cap power as diagnostic tools in specific cancer subtypes to address its real contribution to the biological heterogeneity of human tumors in terms of patient stratification. Several molecular pathways have already been depicted through which p140Cap may exert its tumor suppressing properties, but the extensive analysis of p140Cap interactome in BC cells have recently provided data on its involvement in several additional biological networks relevant for cancer progression. Moreover, p140Cap expression confers at least in NB cells a higher sensitivity to chemotherapeutic drugs, implying its impact on cytoskeletal features and pro-apoptotic processes involved in chemotherapy-induced cell death. In addition, more preclinical work is needed to understand whether p140Cap-expressing tumors might be treated with low doses of drugs or with drug combination regimens. Detailed molecular analysis on these pathways will pave the way for the identification of key p140Cap-dependent mechanisms, including its interacting proteins, as suitable targets for cancer treatment. On the other hand, since many tumors do not express relevant levels of p140Cap, the existing knowledge on miRNAs able to down-regulate p140Cap expression in human tumors, provides the testable hypothesis on the use of specific anti-miRNAs to enhance an appropriate tumor response in preclinical models. It remains to be determined whether p140Cap-regulated pathways might also impact on tumor microenvironment, and on tumor cell metabolism. Overall, the dissection of p140Cap biological features and its regulated pathways in cancer highlight the potential clinical impact of *SRCIN1*/p140Cap expression on patient stratification, as being the relevant key player for patient outcome.

## Abbreviations

*SRCIN1*: Src Kinase Signaling Inhibitor 1
p140Cap: Cap: p130Cas-associated protein
BC: Breast cancer
NB: Neuroblastoma

## References

[1] Chin LS, Nugent RD, Raynor MC, Vavalle JP, Li L (2000). SNIP, a novel SNAP-25-interacting protein implicated in regulated exocytosis. J Biol Chem.

[2] Cabodi S, del Pilar C-L, Di Stefano P, Defilippi P (2010). Integrin signalling adaptors: not only figurants in the cancer story. Nat Rev Cancer.

[3] Di Stefano P, Cabodi S, Boeri Erba E, Margaria V, Bergatto E, Giuffrida MG, Silengo L, Tarone G, Turco E, Defilippi P (2004). P130Cas-associated protein (p140Cap) as a new tyrosine-phosphorylated protein involved in cell spreading. Mol Biol Cell.

[4] Di Stefano P, Leal MP, Tornillo G, Bisaro B, Repetto D, Pincini A, Santopietro E, Sharma N, Turco E, Cabodi S, Defilippi P (2011). The adaptor proteins p140CAP and p130CAS as molecular hubs in cell migration and invasion of cancer cells. Am J Cancer Res.

[5] Repetto D, Aramu S, Boeri Erba E, Sharma N, Grasso S, Russo I, Jensen ON, Cabodi S, Turco E, Di Stefano P, Defilippi P (2013). Mapping of p140Cap phosphorylation sites: the EPLYA and EGLYA motifs have a key role in tyrosine phosphorylation and Csk binding, and are substrates of the Abl kinase. PLoS ONE.

[6] Karasugi T, Semba K, Hirose Y, Kelempisioti A, Nakajima M, Miyake A, Furuichi T, Kawaguchi Y, Mikami Y, Chiba K, Kamata M, Ozaki K, Takahashi A, Makela P, Karppinen J, Kimura T, Kubo T, Toyama Y, Yamamura K, Mannikko M, Mizuta H, Ikegawa S (2009). Association of the tag SNPs in the human SKT gene (KIAA1217) with lumbar disc herniation. J Bone Min Res.

[7] Semba K, Araki K, Li Z, Matsumoto K, Suzuki M, Nakagata N, Takagi K, Takeya M, Yoshinobu K, Araki M, Imai K, Abe K, Yamamura K (2006). A novel murine gene, Sickle tail, linked to the Danforth's short tail locus, is required for normal development of the intervertebral disc. Genetics.

[8] Jaworski J, Kapitein LC, Gouveia SM, Dortland BR, Wulf PS, Grigoriev I, Camera P, Spangler SA, Di Stefano P, Demmers J, Krugers H, Defilippi P, Akhmanova A, Hoogenraad CC (2009). Dynamic microtubules regulate dendritic spine morphology and synaptic plasticity. Neuron.

[9] Repetto D, Camera P, Melani R, Morello N, Russo I, Calcagno E, Tomasoni R, Bianchi F, Berto G, Giustetto M, Berardi N, Pizzorusso T, Matteoli M, Di Stefano P, Missler M, Turco E, Di Cunto F, Defilippi P (2014). p140Cap regulates memory and synaptic plasticity through Src-mediated and citron-N-mediated actin reorganization. J Neurosci.

[10] Li MY, Miao WY, Wu QZ, He SJ, Yan G, Yang Y, Liu JJ, Taketo MM, Yu X (2017) A critical role of presynaptic cadherin/catenin/p140Cap complexes in stabilizing spines and functional synapses in the neocortex. Neuron 94 (6):1155–1172 e1158. doi: 10.1016/j.neuron.2017.05.022 10.1016/j.neuron.2017.05.022 28641114

[11] Di Stefano P, Damiano L, Cabodi S, Aramu S, Tordella L, Praduroux A, Piva R, Cavallo F, Forni G, Silengo L, Tarone G, Turco E, Defilippi P (2007). p140Cap protein suppresses tumour cell properties, regulating Csk and Src kinase activity. EMBO J.

[12] Lamy PJ, Fina F, Bascoul-Mollevi C, Laberenne AC, Martin PM, Ouafik L, Jacot W (2011). Quantification and clinical relevance of gene amplification at chromosome 17q12-q21 in human epidermal growth factor receptor 2-amplified breast cancers. Breast Cancer Res.

[13] Yamauchi M, Sudo K, Ito H, Iwamoto I, Morishita R, Murai T, Kajita K, Ishizuka T, Nagata K (2013). Localization of multidomain adaptor proteins, p140Cap and vinexin, in the pancreatic islet of a spontaneous diabetes mellitus model, Otsuka Long-Evans Tokushima Fatty rats. Med Mol Morphol.

[14] Damiano L, Di Stefano P, Camacho Leal MP, Barba M, Mainiero F, Cabodi S, Tordella L, Sapino A, Castellano I, Canel M, Frame M, Turco E, Defilippi P (2010). p140Cap dual regulation of E-cadherin/EGFR cross-talk and Ras signalling in tumour cell scatter and proliferation. Oncogene.

[15] Grasso S, Chapelle J, Salemme V, Aramu S, Russo I, Vitale N, Verdun di Cantogno L, Dallaglio K, Castellano I, Amici A, Centonze G, Sharma N, Lunardi S, Cabodi S, Cavallo F, Lamolinara A, Stramucci L, Moiso E, Provero P, Albini A, Sapino A, Staaf J, Di Fiore PP, Bertalot G, Pece S, Tosoni D, Confalonieri S, Iezzi M, Di Stefano P, Turco E, Defilippi P (2017). The scaffold protein p140Cap limits ERBB2-mediated breast cancer progression interfering with Rac GTPase-controlled circuitries. Nat Commun.

[16] Ito H, Atsuzawa K, Sudo K, Di Stefano P, Iwamoto I, Morishita R, Takei S, Semba R, Defilippi P, Asano T, Usuda N, Nagata K (2008). Characterization of a multidomain adaptor protein, p140Cap, as part of a pre-synaptic complex. J Neurochem.

[17] Calipari ES, Godino A, Salery M, Damez-Werno DM, Cahill ME, Werner CT, Gancarz AM, Peck EG, Jlayer Z, Rabkin J, Landry JA, Smith ACW, Defilippi P, Kenny PJ, Hurd YL, Neve RL, Dietz DM, Nestler EJ (2019). Synaptic microtubule-associated protein EB3 and SRC phosphorylation mediate structural and behavioral adaptations during withdrawal from cocaine self-administration. J Neurosci.

[18] Damez-Werno DM, Sun H, Scobie KN, Shao N, Rabkin J, Dias C, Calipari ES, Maze I, Pena CJ, Walker DM, Cahill ME, Chandra R, Gancarz A, Mouzon E, Landry JA, Cates H, Lobo MK, Dietz D, Allis CD, Guccione E, Turecki G, Defilippi P, Neve RL, Hurd YL, Shen L, Nestler EJ (2016). Histone arginine methylation in cocaine action in the nucleus accumbens. Proc Natl Acad Sci USA.

[19] Alfieri A, Sorokina O, Adrait A, Angelini C, Russo I, Morellato A, Matteoli M, Menna E, Boeri Erba E, McLean C, Armstrong JD, Ala U, Buxbaum JD, Brusco A, Coute Y, De Rubeis S, Turco E, Defilippi P (2017). Synaptic interactome mining reveals p140Cap as a new hub for PSD proteins involved in psychiatric and neurological disorders. Front Mol Neurosci.

[20] Hayashi K, Suzuki A, Hirai S, Kurihara Y, Hoogenraad CC, Ohno S (2011). Maintenance of dendritic spine morphology by partitioning-defective 1b through regulation of microtubule growth. J Neurosci.

[21] Tomasoni R, Repetto D, Morini R, Elia C, Gardoni F, Di Luca M, Turco E, Defilippi P, Matteoli M (2013). SNAP-25 regulates spine formation through postsynaptic binding to p140Cap. Nat Commun.

[22] Astro V, de Curtis I (2015) Plasma membrane-associated platforms: dynamic scaffolds that organize membrane-associated events. Sci Signal 8 (367):re1. doi: 10.1126/scisignal.aaa3312 10.1126/scisignal.aaa3312 25759479

[23] Wright PE, Dyson HJ (2015). Intrinsically disordered proteins in cellular signalling and regulation. Nat Rev Mol Cell Biol.

[24] Linding R, Jensen LJ, Diella F, Bork P, Gibson TJ, Russell RB (2003). Protein disorder prediction: implications for structural proteomics. Structure.

[25] Cortese MS, Uversky VN, Dunker AK (2008). Intrinsic disorder in scaffold proteins: getting more from less. Prog Biophys Mol Biol.

[26] Ballif BA, Carey GR, Sunyaev SR, Gygi SP (2008). Large-scale identification and evolution indexing of tyrosine phosphorylation sites from murine brain. J Proteome Res.

[27] Sharma N, Repetto D, Aramu S, Grasso S, Russo I, Fiorentino A, Mello-Grand M, Cabodi S, Singh V, Chiorino G, Turco E, Stefano PD, Defilippi P (2013). Identification of two regions in the p140Cap adaptor protein that retain the ability to suppress tumor cell properties. Am J Cancer Res.

[28] Colicelli J (2010) ABL tyrosine kinases: evolution of function, regulation, and specificity. Science signaling 3 (139):re6. doi: 10.1126/scisignal.3139re6 10.1126/scisignal.3139re6 PMC2954126 20841568

[29] Hernandez SE, Krishnaswami M, Miller AL, Koleske AJ (2004). How do Abl family kinases regulate cell shape and movement?. Trends Cell Biol.

[30] Mimuro H, Suzuki T, Tanaka J, Asahi M, Haas R, Sasakawa C (2002). Grb2 is a key mediator of helicobacter pylori CagA protein activities. Mol Cell.

[31] Hatakeyama M (2004). Oncogenic mechanisms of the Helicobacter pylori CagA protein. Nat Rev Cancer.

[32] Hatakeyama M (2008). Linking epithelial polarity and carcinogenesis by multitasking Helicobacter pylori virulence factor CagA. Oncogene.

[33] Safari F, Murata-Kamiya N, Saito Y, Hatakeyama M (2011). Mammalian Pragmin regulates Src family kinases via the Glu-Pro-Ile-Tyr-Ala (EPIYA) motif that is exploited by bacterial effectors. Proc Natl Acad Sci USA.

[34] Latour S, Veillette A (2001). Proximal protein tyrosine kinases in immunoreceptor signaling. Curr Opin Immunol.

[35] Damiano L, Le Devedec SE, Di Stefano P, Repetto D, Lalai R, Truong H, Xiong JL, Danen EH, Yan K, Verbeek FJ, De Luca E, Attanasio F, Buccione R, Turco E, van de Water B, Defilippi P (2012) p140Cap suppresses the invasive properties of highly metastatic MTLn3-EGFR cells via impaired cortactin phosphorylation. Oncogene 31 (5):624-633. doi:10.1038/onc.2011.257 10.1038/onc.2011.257 21725361

[36] McFarland MA, Ellis CE, Markey SP, Nussbaum RL (2008). Proteomics analysis identifies phosphorylation-dependent alpha-synuclein protein interactions. Mol Cell Proteomics.

[37] Ballif BA, Cao Z, Schwartz D, Carraway KL, Gygi SP (2006). Identification of 14–3-3ε substrates from embryonic murine brain. J Proteome Res.

[38] Lenihan JA, Saha O, Heimer-McGinn V, Cryan JF, Feng G, Young PW (2017). Decreased anxiety-related behaviour but apparently unperturbed NUMB function in ligand of NUMB protein-X (LNX) 1/2 double knockout mice. Mol Neurobiol.

[39] Wilkinson B, Li J, Coba MP (2017). Synaptic GAP and GEF complexes cluster proteins essential for GTP signaling. Sci Rep.

[40] Huttlin EL, Bruckner RJ, Paulo JA, Cannon JR, Ting L, Baltier K, Colby G, Gebreab F, Gygi MP, Parzen H, Szpyt J, Tam S, Zarraga G, Pontano-Vaites L, Swarup S, White AE, Schweppe DK, Rad R, Erickson BK, Obar RA, Guruharsha KG, Li K, Artavanis-Tsakonas S, Gygi SP, Harper JW (2017). Architecture of the human interactome defines protein communities and disease networks. Nature.

[41] Huttlin EL, Ting L, Bruckner RJ, Gebreab F, Gygi MP, Szpyt J, Tam S, Zarraga G, Colby G, Baltier K, Dong R, Guarani V, Vaites LP, Ordureau A, Rad R, Erickson BK, Wuhr M, Chick J, Zhai B, Kolippakkam D, Mintseris J, Obar RA, Harris T, Artavanis-Tsakonas S, Sowa ME, De Camilli P, Paulo JA, Harper JW, Gygi SP (2015). The BioPlex network: a systematic exploration of the human interactome. Cell.

[42] Giurato G, Nassa G, Salvati A, Alexandrova E, Rizzo F, Nyman TA, Weisz A, Tarallo R (2018). Quantitative mapping of RNA-mediated nuclear estrogen receptor beta interactome in human breast cancer cells. Sci Data.

[43] Cancer Genome Atlas N (2012). Comprehensive molecular portraits of human breast tumours. Nature.

[44] Siegel R, DeSantis C, Virgo K, Stein K, Mariotto A, Smith T, Cooper D, Gansler T, Lerro C, Fedewa S, Lin C, Leach C, Cannady RS, Cho H, Scoppa S, Hachey M, Kirch R, Jemal A, Ward E (2012) Cancer treatment and survivorship statistics, 2012. CA 62 (4):220–241. doi: 10.3322/caac.21149 10.3322/caac.21149 22700443

[45] Brodeur GM (2003). Neuroblastoma: biological insights into a clinical enigma. Nat Rev Cancer.

[46] Louis CU, Shohet JM (2015). Neuroblastoma: molecular pathogenesis and therapy. Annu Rev Med.

[47] Grasso S, Cangelosi D, Chapelle J, Alzona M, Centonze G, Lamolinara A, Salemme V, Angelini C, Morellato A, Saglietto A, Bianchi FT, Cabodi S, Salaroglio IC, Fusella F, Ognibene M, Iezzi M, Pezzolo A, Poli V, Di Cunto F, Eva A, Riganti C, Varesio L, Turco E, Defilippi P (2020). Correction to: The SRCIN1/p140Cap adaptor protein negatively regulates the aggressiveness of neuroblastoma. Cell Death Differ.

[48] Monclair T, Brodeur GM, Ambros PF, Brisse HJ, Cecchetto G, Holmes K, Kaneko M, London WB, Matthay KK, Nuchtern JG, von Schweinitz D, Simon T, Cohn SL, Pearson AD, Force IT (2009). The international neuroblastoma risk group (INRG) staging system: an INRG task force report. J Clin Oncol.

[49] Staaf J, Jonsson G, Ringner M, Vallon-Christersson J, Grabau D, Arason A, Gunnarsson H, Agnarsson BA, Malmstrom PO, Johannsson OT, Loman N, Barkardottir RB, Borg A (2010). High-resolution genomic and expression analyses of copy number alterations in HER2-amplified breast cancer. Breast Cancer Res.

[50] Bown N, Cotterill S, Lastowska M, O'Neill S, Pearson AD, Plantaz D, Meddeb M, Danglot G, Brinkschmidt C, Christiansen H, Laureys G, Speleman F, Nicholson J, Bernheim A, Betts DR, Vandesompele J, Van Roy N (1999). Gain of chromosome arm 17q and adverse outcome in patients with neuroblastoma. N Engl J Med.

[51] Chou TC (2010). Drug combination studies and their synergy quantification using the Chou-Talalay method. Can Res.

[52] Vandesompele J, Baudis M, De Preter K, Van Roy N, Ambros P, Bown N, Brinkschmidt C, Christiansen H, Combaret V, Lastowska M, Nicholson J, O'Meara A, Plantaz D, Stallings R, Brichard B, Van den Broecke C, De Bie S, De Paepe A, Laureys G, Speleman F (2005). Unequivocal delineation of clinicogenetic subgroups and development of a new model for improved outcome prediction in neuroblastoma. J Clin Oncol.

[53] Gross N, Beck D, Portoukalian J, Favre S, Carrel S (1989). New anti-GD2 monoclonal antibodies produced from gamma-interferon-treated neuroblastoma cells. Int J Cancer.

[54] Di Leva G, Garofalo M, Croce CM (2014). MicroRNAs in cancer. Ann Rev Pathol.

[55] Orso F, Quirico L, Dettori D, Coppo R, Virga F, Ferreira LC, Paoletti C, Baruffaldi D, Penna E, Taverna D (2020). Role of miRNAs in tumor and endothelial cell interactions during tumor progression. Semin Cancer Biol.

[56] Cao M, Hou D, Liang H, Gong F, Wang Y, Yan X, Jiang X, Wang C, Zhang J, Zen K, Zhang CY, Chen X (2014). miR-150 promotes the proliferation and migration of lung cancer cells by targeting SRC kinase signalling inhibitor 1. Eur J Cancer.

[57] Chen R, Liao JY, Huang J, Chen WL, Ma XJ, Luo XD (2018). Downregulation of SRC kinase signaling inhibitor 1 (SRCIN1) expression by MicroRNA-32 promotes proliferation and epithelial-mesenchymal transition in human liver cancer cells. Oncol Res.

[58] Gao Y, Xue Q, Wang D, Du M, Zhang Y, Gao S (2015). miR-873 induces lung adenocarcinoma cell proliferation and migration by targeting SRCIN1. Am J Trans Res.

[59] Li N, Piao J, Wang X, Kim KY, Bae JY, Ren X, Lin Z (2019). Paip1 indicated poor prognosis in cervical cancer and promoted cervical carcinogenesis. Cancer Res Treatment.

[60] Liu L, Wang W, Gao S, Wang X (2019). MicroRNA208a directly targets Src kinase signaling inhibitor 1 to facilitate cell proliferation and invasion in nonsmall cell lung cancer. Mol Med Rep.

[61] Ma L, Shao Z, Zhao Y (2019). MicroRNA-374a promotes pancreatic cancer cell proliferation and epithelial to mesenchymal transition by targeting SRCIN1. Pathol Res Pract.

[62] Quan X, Chen D, Li M, Chen X, Huang M (2019). MicroRNA-150-5p and SRC kinase signaling inhibitor 1 involvement in the pathological development of gastric cancer. Exp Therapeutic Med.

[63] Wu W, He L, Huang Y, Hou L, Zhang W, Zhang L, Wu C (2019). MicroRNA-510 plays oncogenic roles in non-small cell lung cancer by directly targeting SRC kinase signaling inhibitor 1. Oncol Res.

[64] Xu X, Wang W, Su N, Zhu X, Yao J, Gao W, Hu Z, Sun Y (2015). miR-374a promotes cell proliferation, migration and invasion by targeting SRCIN1 in gastric cancer. FEBS Lett.

[65] Ye L, Wang H, Liu B (2016). miR-211 promotes non-small cell lung cancer proliferation by targeting SRCIN1. Tumour Biol.

[66] Zhang L, Lin J, Ye Y, Oba T, Gentile E, Lian J, Wang J, Zhao Y, Gu J, Wistuba II, Roth JA, Ji L, Wu X (2018). Serum MicroRNA-150 Predicts Prognosis for early-stage non-small cell lung cancer and promotes tumor cell proliferation by targeting tumor suppressor gene SRCIN1. Clin Pharmacol Ther.

[67] Zhou P, Xiong T, Yao L, Yuan J (2020). MicroRNA-665 promotes the proliferation of ovarian cancer cells by targeting SRCIN1. Exp Therapeutic Med.

[68] Zhu J, Han S (2019). miR-150-5p promotes the proliferation and epithelial-mesenchymal transition of cervical carcinoma cells via targeting SRCIN1. Pathol Res Pract.

[69] Sun W, Wang X, Li J, You C, Lu P, Feng H, Kong Y, Zhang H, Liu Y, Jiao R, Chen X, Ba Y (2018). MicroRNA-181a promotes angiogenesis in colorectal cancer by targeting SRCIN1 to promote the SRC/VEGF signaling pathway. Cell Death Dis.

[70] Lu Q, Guo Z, Qian H (2019). Role of microRNA-150-5p/SRCIN1 axis in the progression of breast cancer. Exp Therapeutic Med.

[71] Bai PS, Hou P, Kong Y (2018). Hepatitis B virus promotes proliferation and metastasis in male Chinese hepatocellular carcinoma patients through the LEF-1/miR-371a-5p/SRCIN1/pleiotrophin/Slug pathway. Exp Cell Res.

[72] Mailleux AA, Overholtzer M, Schmelzle T, Bouillet P, Strasser A, Brugge JS (2007). BIM regulates apoptosis during mammary ductal morphogenesis, and its absence reveals alternative cell death mechanisms. Dev Cell.

[73] Laurin M, Huber J, Pelletier A, Houalla T, Park M, Fukui Y, Haibe-Kains B, Muller WJ, Cote JF (2013). Rac-specific guanine nucleotide exchange factor DOCK1 is a critical regulator of HER2-mediated breast cancer metastasis. Proc Natl Acad Sci USA.

[74] Wertheimer E, Gutierrez-Uzquiza A, Rosemblit C, Lopez-Haber C, Sosa MS, Kazanietz MG (2012). Rac signaling in breast cancer: a tale of GEFs and GAPs. Cell Signal.

[75] Chapelle J, Baudino A, Torelli F, Morellato A, Angelini C, Salemme V, Centonze G, Natalini D, Gai M, Savino A, Poli V, Turco E, Defilippi P (2020) The N-terminal domain of the adaptor protein p140Cap interacts with Tiam1 and controls Tiam1/Rac1 axis. American journal of cancer research, in press. PMC7783762 33415001

[76] Bill R, Christofori G (2015). The relevance of EMT in breast cancer metastasis: correlation or causality?. FEBS Lett.

[77] Lamouille S, Xu J, Derynck R (2014). Molecular mechanisms of epithelial-mesenchymal transition. Nat Rev Mol Cell Biol.

[78] Kratimenos P, Koutroulis I, Marconi D, Syriopoulou V, Delivoria-Papadopoulos M, Chrousos GP, Theocharis S (2014). Multi-targeted molecular therapeutic approach in aggressive neuroblastoma: the effect of Focal Adhesion Kinase-Src-Paxillin system. Expert Opin Therapeutic Targets.

[79] Navarra M, Celano M, Maiuolo J, Schenone S, Botta M, Angelucci A, Bramanti P, Russo D (2010). Antiproliferative and pro-apoptotic effects afforded by novel Src-kinase inhibitors in human neuroblastoma cells. BMC cancer.

[80] Radi M, Brullo C, Crespan E, Tintori C, Musumeci F, Biava M, Schenone S, Dreassi E, Zamperini C, Maga G, Pagano D, Angelucci A, Bologna M, Botta M (2011). Identification of potent c-Src inhibitors strongly affecting the proliferation of human neuroblastoma cells. Bioorg Med Chem Lett.

[81] Ara T, Nakata R, Sheard MA, Shimada H, Buettner R, Groshen SG, Ji L, Yu H, Jove R, Seeger RC, DeClerck YA (2013). Critical role of STAT3 in IL-6-mediated drug resistance in human neuroblastoma. Can Res.

[82] Avalle L, Camporeale A, Camperi A, Poli V (2017). STAT3 in cancer: a double edged sword. Cytokine.

[83] Odate S, Veschi V, Yan S, Lam N, Woessner R, Thiele CJ (2017) Inhibition of STAT3 with the generation 2.5 antisense oligonucleotide, azd9150, decreases neuroblastoma tumorigenicity and increases chemosensitivity. Clin Cancer Res 23 (7):1771–1784. doi: 10.1158/1078-0432.CCR-16-1317 10.1158/1078-0432.CCR-16-1317 PMC5381521 27797972

[84] Turinetto V, Giachino C (2015). Multiple facets of histone variant H2AX: a DNA double-strand-break marker with several biological functions. Nucl Acids Res.

[85] Chapelle J, Sorokina O, McLean C, Salemme V, Alfieri A, Angelini C, Morellato A, Adrait A, Menna E, Matteoli M, Coute Y, Ala U, Turco E, Defilippi P, Armstrong JD (2019). Dissecting the Shared and context-dependent pathways mediated by the p140Cap adaptor protein in cancer and in neurons. Front Cell Dev Biol.

[86] Basu S, Cheriyamundath S, Ben-Ze'ev A (2018) Cell-cell adhesion: linking Wnt/beta-catenin signaling with partial EMT and stemness traits in tumorigenesis. F1000Research 7. doi:10.12688/f1000research.15782.1 10.12688/f1000research.15782.1 PMC6144947 30271576

[87] Bagnato P, Castagnino A, Cortese K, Bono M, Grasso S, Bellese G, Daniele T, Lundmark R, Defilippi P, Castagnola P, Tacchetti C (2017). Cooperative but distinct early co-signaling events originate from ERBB2 and ERBB1 receptors upon trastuzumab treatment in breast cancer cells. Oncotarget.

